# Natural Variation of Vitamin D and Neurofilament Light Chain in Relapsing-Remitting Multiple Sclerosis

**DOI:** 10.3389/fneur.2020.00329

**Published:** 2020-04-30

**Authors:** Egil Røsjø, Jonas C. Lindstrøm, Trygve Holmøy, Kjell-Morten Myhr, Kristin N. Varhaug, Øivind Torkildsen

**Affiliations:** ^1^Volvat Medical Center, Oslo, Norway; ^2^Institute of Clinical Medicine, University of Oslo, Oslo, Norway; ^3^Health Services Research Unit, Akershus University Hospital, Lørenskog, Norway; ^4^Department of Neurology, Akershus University Hospital, Lørenskog, Norway; ^5^Department of Clinical Medicine, University of Bergen, Bergen, Norway; ^6^Neuro-SysMed, Department of Neurology, Haukeland University Hospital, Bergen, Norway

**Keywords:** relapsing-remitting multiple sclerosis, 25-hydroxyvitamin D, neurofilament light chain, magnetic resonance imaging, interferon-β

## Abstract

**Background:** High serum levels of 25-hydroxyvitamin D (25(OH)D) have been found among patients with a favorable disease course in relapsing-remitting MS (RRMS), indicating that this may limit clinical deterioration. Clinical deterioration in RRMS correlates with increasing serum levels of neurofilament light chain (NfL).

**Objectives:** To examine the association between physiological variations in serum 25(OH)D and NfL levels in RRMS patients before and during disease modifying therapy (DMT).

**Material and Methods:** Serum 25(OH)D and NfL concentrations were measured in 85 newly diagnosed RRMS patients enrolled in a 24-month randomized double-blinded placebo-controlled trial of ω-3 fatty acid supplementation without vitamin D. Patients were without DMT until interferon β-1a (IFN-β) initiation at study month 6. Longitudinal serum measurements and brain magnetic resonance imaging (MRI) were obtained. Associations between 25(OH)D and NfL levels were analyzed with linear regression models for the whole study period and the periods before and during IFN-β treatment. Analyses with adjustment for inflammatory MRI disease activity were also performed.

**Results:** No significant associations were found between variations in 25(OH)D and NfL levels during the whole study period (*p* = 0.95), or the periods without (*p* = 0.78) or with (*p* = 0.33) IFN-β therapy. Patients with inflammatory MRI disease activity had significantly higher serum NfL levels than patients without inflammatory MRI disease activity [mean (SD) difference 12.6 (2.0) pg/mL, *p* < 0.01]. Adjustment for this did not change the relationship between 25(OH)D and NfL concentrations.

**Conclusion:** Natural variations in serum 25(OH)D values do not seem to be associated with alterations in serum NfL concentrations in RRMS patients.

## Introduction

Central nervous tissue degeneration promotes disease progression in multiple sclerosis (MS), and neurofilament light chain (NfL) is a validated surrogate biomarker for this process in patients with relapsing-remitting MS (RRMS) (1). NfL is a major component of the cytoskeleton in neuronal axons and levels of NfL in cerebrospinal fluid (CSF) and blood reflect axonal loss in RRMS from an early disease stage (2). Moreover, high serum concentrations of NfL have been associated with inflammatory disease activity and development of atrophy on brain magnetic resonance imaging (MRI) and clinical disability (3). Disease modifying therapies (DMTs) reduce NfL levels in RRMS patients, but it is currently unclear if this is due to their anti-inflammatory properties, direct effect on neurodegeneration or a combination of these actions (4, 5).

Naturally increasing serum levels of 25(OH)D have been associated with decreased clinical and inflammatory MRI disease activity in observational studies in RRMS (6, 7). Furthermore, a good vitamin D status has been found to be associated with low levels of NfL in CSF from MS patients in an observational setting (8).

Results from randomized placebo-controlled trials (RCTs) have indicated a relationship between increasing serum levels of 25(OH)D after vitamin D supplementation and reduced inflammatory MRI disease activity in RRMS (9). However, the effect of vitamin D treatment on serum levels of NfL is uncertain, as it has only been investigated in RCTs with a limited number of rather clinically stable patients (10, 11).

Vitamin D intervention trials have not been able to obtain MRI or clinical results in the magnitude of what was indicated by earlier observational studies (6, 7). The natural variation in serum 25(OH)D concentrations in observational studies is largely driven by seasonal differences in ultraviolet radiation (UVR) exposure (12), and it has been speculated that discrepancies between the results from observational studies of vitamin D and RCTs of vitamin D supplementation in RRMS may among other be related to UVR exposure having beneficial effects beyond increasing the 25(OH)D serum level (9). It is therefore pertinent to explore if observed natural variations in serum 25(OH)D levels are related to neurodegeneration, as measured by change in serum NfL levels, in RRMS.

We have previously reported results from a two-year observational study of 85 RRMS patients with rather high disease activity showing an inverse association between naturally increasing 25(OH)D levels and inflammatory MRI disease activity before introduction of interferon beta-1a (IFN-β) treatment (6), while NfL levels were positively correlated with inflammatory MRI disease activity and inversely with IFN-β initiation (5). However, in a two-year double-blinded RCT of high-dose oral vitamin D_3_ supplementation (20,000 IU/week) in another cohort of 68 patients with RRMS with lower disease activity we found only a strong trend for a reduction among patients without DMTs and no overall effect of increasing serum 25(OH)D levels on serum levels of NfL (10). The aim of the current study was therefore to clarify the potential relationship between natural variations in 25(OH)D serum concentrations and change in serum NfL levels both prior to and during IFN-β therapy in our observational RRMS cohort.

## Materials and Methods

### Trial Cohort and Design

Study details and clinical and MRI results have been published earlier (13). Briefly, 92 Norwegian RRMS patients with age 18–55 years, EDSS score ≤5, no use of DMTs the last 6 months, and either ≥1 clinical relapse, new T1-weigheted gadolinium-enhancing (T1 Gd^+^) or new or enlarging T2-weigheted MRI lesion the last 12 months before baseline were included and followed for 24 months. Inclusion was continuous throughout the year from December 2004 to July 2006 and the patients were allocated 1:1 to daily ω-3 (Triomar, Pronova Biocare AS, Sandefjord, Norway) or placebo capsules both without vitamin D for 24 months. Treatment with thrice-weekly 44 μg subcutaneous IFN-β (Rebif, Merck Serono, Geneva, Switzerland) was initiated at study month 6 for all and continued throughout the remaining 18 months of the trial for all but 5 patients. No other DMTs were used during the study. The Regional Committee for Medical and Health Research Ethics in Western Norway and the Norwegian Medicines Agency approved the study protocol, written informed consent was obtain from all patients before study-enrollment and the study was registered at clinicaltrails.gov (NCT00360906).

### Laboratory, Clinical and MRI Measurements

Serum samples were available for measurements from baseline and study months 3, 6, 12, and 24 for both NfL and 25(OH)D, and in addition at months 1, 7, 9, and 18 for 25(OH)D. Concentrations of 25(OH)D were determined by a radioimmunoassay and NfL with a Simoa assay, as described earlier (6, 14). Brain MRIs were acquired monthly the first nine months and at months 12 and 24, as previously described (13). Inflammatory MRI disease activity in the current study was defined by the presence of new T1 Gd^+^ lesions.

### Missing Data and Statistical Analyses

Eighty-eight of the original 92 enrolled patients were included in this study, and 3 of these patients only contributed with 25(OH)D and MRI measurements. Among the 85 patients with NfL measurements, 10 had one missing value. Twenty-three out of a possible total of 1020 scans were missing, with only one patient having <10 scans. Baseline associations and correlations between 25(OH)D and NfL and potential confounding factors were analyzed with independent samples *t*-tests and Pearson correlations. Comparisons between mean values at baseline and end of study were examined with paired sample *t*-tests. Associations between paired 25(OH)D and NfL levels and NfL levels lagged 3 months behind 25(OH)D levels were analyzed with linear regression models with random intercepts for each patient for the whole study period and the periods without and with IFN-β treatment. Models with adjustment for the effect of MRI disease activity on NfL levels were calculated in additional analyses. The intraclass correlation coefficient (ICC) for the NfL measurements was calculated with a random effects model with random intercepts for each patient. The analyses were performed with IBM SPSS Statistics version 25 and R with the lme4 package.

## Results

### Baseline Characteristics

Earlier reported baseline gender distribution, age, disease duration, EDSS score, HLA-DRB1^*^15 status and baseline 25(OH)D serum values (6), and body mass index (BMI) and NfL serum levels are included together in Table 1. We have previously found a positive correlation between baseline 25(OH)D and NfL levels and a negative correlation between BMI and NfL measurements (10), but no similar correlations or other associations or correlations were found between these characteristics in the current cohort (data not shown). Still, high levels of NfL were noted both at lower and higher levels of 25(OH)D (Supplemental figure 1).

**Table 1:** Baseline characteristics.

Gender (women-men); *N*	57–31
Age (years); mean (SD)	38.9 (8.3)
Disease duration (years); mean (SD)	1.9 (3.1)
EDSS score; median (IQR)	2.0 (1.5–2.5)
HLA-DRB1*15 status (positive-negative); *N*	58–26^†^
BMI; mean (SD)	25.7 (4.3)
25(OH)D (nmol/L); mean (SD)	61.2 (19.3)^‡^
NfL (pg/mL); mean (SD)	40.2 (22.4)^*^

*N, Number; SD, Standard deviation; IQR, Inter quartile range; EDSS, Kurtzke Expanded Disability Status Scale; BMI, Body mass index; 25(OH)D, 25-hydroxyvitamin D; NfL, Neurofilament light chain*.

† *4 missing values*.

‡ *1 missing value*.

* *3 missing values*.

### Relationship Between Serum Levels of 25(OH)D and NfL

**Figure 1:**
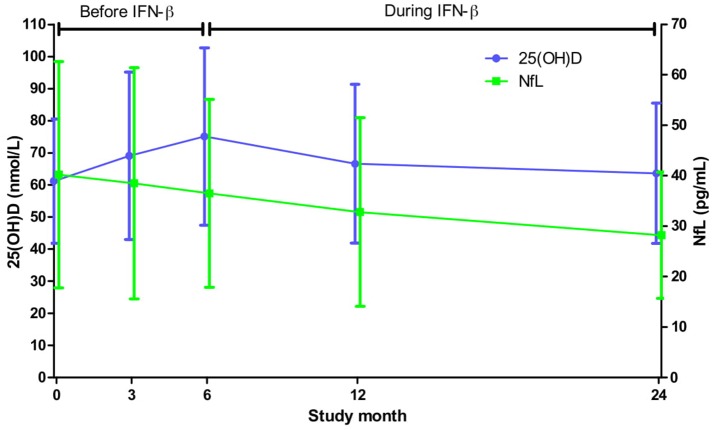
Mean (SD) serum levels of 25(OH)D and NfL throughout the study. Based on all available 25(OH)D and NfL measurements from a minimum of 85 and 79 patients, respectively, at each point of time.

Although high-dose oral vitamin D supplementation has not shown an effect on serum NfL levels in RRMS (10, 11), we wanted to examine if there was a relationship between serum 25(OH) and NfL concentrations in a setting where change in 25(OH)D levels were mainly driven by seasonal variation in UVR exposure. Previously reported 25(OH)D and NfL serum concentrations during the 24-month study period are shown together in Figure 1 and Supplemental Table 1 (5, 6). As the measurements were obtained within the same season, no significant change was found between baseline and end of study values of 25(OH)D. However, the NfL concentrations were significantly reduced from study initiation to conclusion [mean (SD) level of 40.2 (22.4) and 28.2 (12.5) pg/mL, respectively, *p* < 0.01].

**Figure 2:**
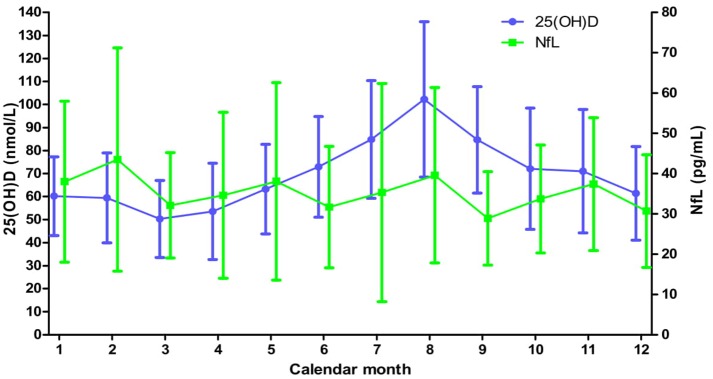
Mean (SD) serum levels of 25(OH)D and NfL levels throughout the calendar year for the whole study. Based on all available 25(OH)D and NfL measurements from a minimum of 41 and 16 patients, respectively, at each month.

Due to the northern location of Norway, vitamin D status varies substantially between winter (November to March) and summer (May to September). Figure 2 and Supplemental Table 2 show the serum 25(OH)D and NfL concentrations from the whole study period stratified by calendar month. The peak 25(OH)D level was found in August [mean (SD) 102.3 (33.7) nmol/L] and the lowest value was seen in March [mean (SD) 50.3 (16.8) nmol/L], while the highest NfL level was in February [mean (SD) 43.5 (27.7) pg/mL] and at the bottom level in September [mean (SD) 28.9 (11.6) pg/mL]. However, no significant association was found when analyzing paired 25(OH)D and NfL measurements for the whole study period with a linear regression model [decrease of NfL of 0.1% (95% CI −1.8 −1.7%) with each 10 nmol/L increase of 25(OH)D, *p* = 0.95].

**Figure 3:**
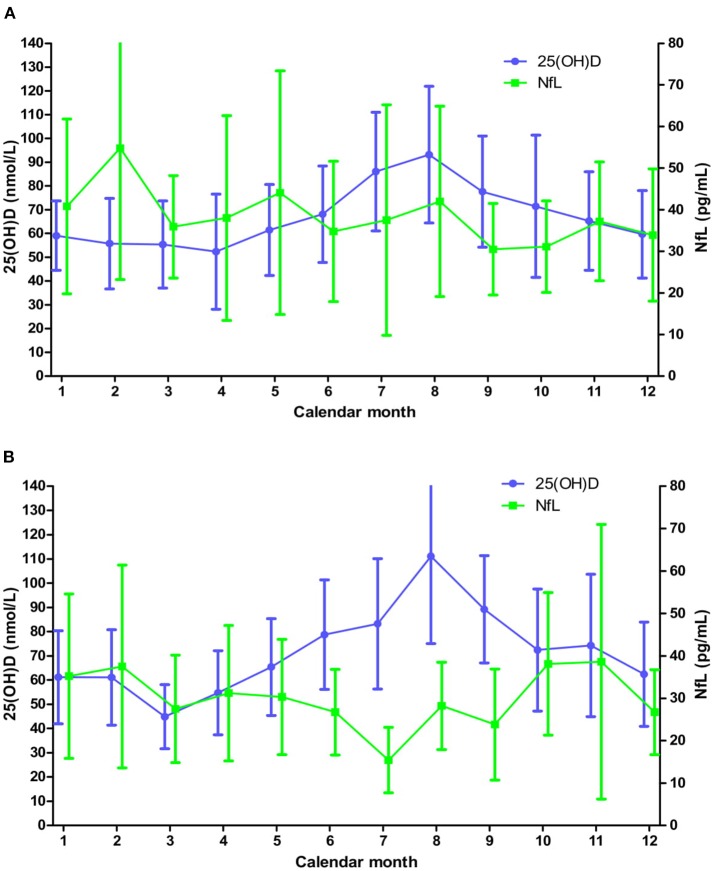
Mean (SD) serum levels of 25(OH)D and NfL levels throughout the calendar year before **(A)** and during **(B)** IFN-β treatment. **(A)** Based on all available 25(OH)D and NfL measurements from a minimum of 14 and 10 patients, respectively, at each month. **(B)** Based on all available 25(OH)D and NfL measurements from a minimum of 18 and 2 patients, respectively, at each month.

We have earlier seen a trend for a reduction in serum NfL levels among RRMS patients without DMTs on vitamin D supplementation (10). Moreover, we have shown that introduction of IFN-β therapy reduces NfL levels in the current patient cohort (5). We therefore examined associations between 25(OH)D and NfL concentrations in the period before (study month 0–6) and during (study month 7–24) IFN-β treatment separately (Figure 3 and Supplemental Tables 3, 4). 25(OH)D levels had expected seasonal variations and again the highest value of NfL was found in winter [54.8 (31.6) pg/mL in February prior to and 38.6 (32.4) pg/mL in November during IFN-β therapy] and the lowest in summer [30.5 (11.0) pg/mL in September prior to and 15.4 (7.7) in July during IFN-β therapy]. Nevertheless, no associations were found between change in 25(OH)D and NfL levels when the different study periods were analyzed with linear regression models of paired samples (Table 2).

**Table 2:** Change in serum NfL concentration with every 10 nmol/L increase of 25(OH)D.

**Study period**	% (CI)^*^	*p*-value^*^
Before IFN-β	−0.3 (−2.0–1.5)	0.78
During IFN-β	1.6 (−1.6–4.8)	0.33

*IFN-β, Interferon beta-1a; CI, 95% confidence interval*.

* *Calculated with linear regression models with random intercepts for each patient and inclusion of only paired 25(OH)D and NfL measurements*.

### Effect of MRI Activity on the Relationship Between Serum Levels of 25(OH)D and NfL

**Figure 4:**
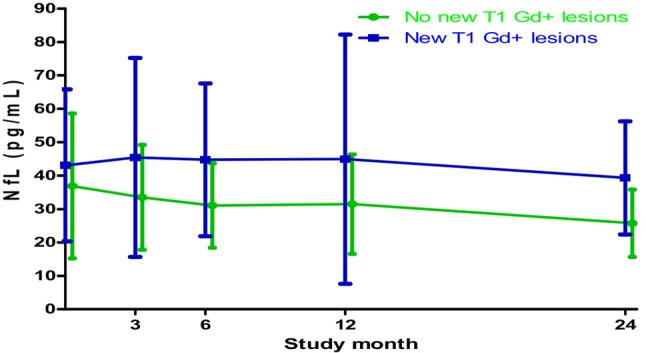
Mean (SD) serum NfL levels in patients with and without new T1-weighted gadolinium-enhancing (T1 Gd+) lesions on MRI scans throughout the study. Based on all available NfL measurements from a minimum of 79 patients at each point of time.

Serum concentration of NfL rise with MRI disease activity in RRMS patients (3, 5), and a significantly higher average NfL concentration was found among patients with inflammatory MRI disease activity (T1 Gd+ scans) compared to those without such MRI activity during the study [mean difference (SD) 12.6 (2.0) pg/mL, *p* < 0.01] (Figure 4). In order to examine if changes in 25(OH)D levels were associated with alterations in NfL levels independently of inflammatory activity, we performed additional analyses of the relationship between the serum 25(OH)D and NfL measurements with adjustment for the effect of MRI disease activity on the NfL levels. However, this adjustment had no clear effect, as no significant associations were seen between change in 25(OH)D levels and NfL concentrations during the whole study period nor in the periods stratified by IFN-β use (Table 3).

**Table 3:** Change in serum NfL concentration with every 10 nmol/L increase of 25(OH)D when controlling for MRI disease activity.

**Study period**	% (CI)^*^	*p*-value^*^
Whole	0.6 (−1.1–2.2)	0.49
Before IFN-β	0.1 (−1.8–1.9)	0.95
During IFN-β	1.4 (−1.8–4.6)	0.38

*IFN-β, Interferon beta-1a; CI, 95% confidence interval*.

* *Calculated with linear regression models with random intercepts for each patient, adjustment for T1-weigheted gadolinium-enhancing lesion activity on MRI and inclusion of only paired 25(OH)D and NfL measurements*.

## Discussion

This study explored the relationship between serum 25(OH)D and NfL in RRMS in an observational setting, and is to our knowledge the first longitudinal study examining the potential relation of naturally varying 25(OH)D levels with NfL concentrations in blood from RRMS patients with and without DMT. The main result of the study is negative, as we found no significant association between increasing levels of 25(OH)D and change in NfL levels in serum in the two-year study period. Likewise, no relationships were found in the period before or after initiation of IFN-β treatment, nor when controlling for the effect of inflammatory MRI disease activity on the NfL concentration.

A good vitamin D status has earlier been associated with both reduced MRI disease activity and clinical progression among RRMS patients on IFN-β treatment in an observational setting (15). However, our results do not support an effect of rising 25(OH)D levels driven mainly by seasonal alterations in UVR exposure on axonal degeneration in RRMS, as measured by serum NfL levels. This is in line with the finding of no effect of high-dose oral vitamin D_3_ supplementation on serum NfL levels in RCTs among RRMS patients with DMT (10, 11).

**Figure 5:**
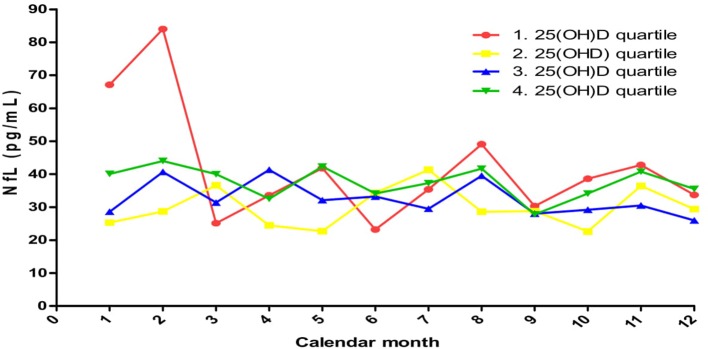
Mean serum NfL concentrations during the year when stratified by 25(OH)D quartiles based on whole study mean values 1. quartile: 30.9–55.6 nmol/L; 2. quartile: 56.1–65.8 nmol/L; 3. quartile: 66.2–78.3 nmol/L;4. quartile: 78.4–128.6 nmol/L.

We previously reported a positive correlation between baseline 25(OH)D and NfL serum levels that was not confirmed in this patient cohort (10). However, we found high NfL levels with both low and high 25(OH)D levels. Moreover, when including measurements from the whole study and stratifying patients into quartiles by their mean 25(OH)D level, the first and fourth quartile had the highest average NfL levels at most time points throughout the year (Figure 5). This may support earlier observational data suggesting a clinical benefit in improvement from a poor [25(OH)D < 25 nmol/L) to a sufficient (25(OH)D ~ 50 nmol/L] vitamin D status (15), while an uncertain clinical benefit of vitamin D supplementation has been found in RCTs with an improvement from a sufficient to a good [25(OH)D > 75 nmol/L] vitamin D status (9). However, we and others did not find an increase in NfL concentration in patients reaching high 25(OH)D levels during vitamin D intervention trials (10, 11).

The negative results reported herein may be due to several different reasons. Although the 25(OH)D level in serum rise rather quickly during summer and it is expected that levels in the central nervous system will also increase within a rather short time span (16), a possible positive effect of this on neurodegeneration and a subsequent drop in serum NfL may be more protracted. This may explain why we failed to find a significant association between rising 25(OH)D and change in NfL concentration during the six-month period without DMT. However, the serum NfL levels seemed to decrease rather quickly during summer, and no relationship was found when analyzing the association between changes in 25(OH)D and NfL levels with NfL measurements lagged three months behind 25(OH)D measurements (Supplemental Table 5). Moreover, we have earlier shown a significant reduction in the serum NfL concentration after IFN-β introduction in this patient cohort (5). This may have overshadowed a limited relationship between 25(OH)D and NfL levels during IFN-β therapy, similarly to what was seen in our earlier report on serum inflammation markers among these patients (17). Furthermore, although a negative association between increasing 25(OH)D levels and inflammatory MRI disease activity and a positive association between inflammatory MRI disease activity and NfL levels have been reported in this cohort (5, 6), it is clear that axonal degeneration is not only driven by neruoinflammation (18). Lastly, it may be speculated that inter-individual differences in serum levels of 25(OH)D at a given time point are related to differing inflammatory disease activity (9), and that seasonal variation in inflammatory MRI disease activity and NfL levels are due to seasonal beneficial factors for both neuroinflammation and neurodegeneration not related to 25(OH)D or UVR exposure (9, 19).

The current study has several limitations. Firstly, the total number of enrolled patients and NfL measurements were limited when taking into account that half of the total variation of the NfL levels was inter-individual (ICC of 0.50). Secondly, the 25(OH)D levels of the patients were rather high throughout the year with mean concentrations being over 50 nmol/L every month. Thirdly, the period of time the patients were without DMT was short, and even though the time interval between serum measurements during DMT treatment was quite long we cannot rule out that changes in serum 25(OH)D levels may be related to alternations in NfL serum concentrations beyond the timeframe of this study. However, the belief in the main result may be strengthened by the study design and its stringent conduction, as it was originally designed as a RCT of ω-3 supplementation. Specifically, it should be noted that the continuous enrollment throughout the year lead to a quite even amount of measurements being obtained during winter and summer time. Moreover, the strength of the results is increased by the consistent outcome in the overall and sub-period analyses, and in the adjusted analyses.

Although an increase in serum 25(OH)D levels may reduce short-term MRI activity among vitamin D sufficient RRMS patients, it is still unclear if this has a long-term effect on axonal degeneration and disability progression (9). Furthermore, the positive effect of a high 25(OH)D level on brain atrophy in an observational setting seems to be in conflict with no reduction in serum NfL levels in RCTs of vitamin D supplementation (10, 11, 15). It is therefore advisable that further studies on 25(OH)D and NfL should be carried out in short-term RCTs with vitamin D supplementation as an add-on to other DMTs in patients with a poor vitamin D status, and in larger and more long-term observational studies among patients that are vitamin D sufficient.

In conclusion, the results from this observational study do not support a significant association between naturally increasing 25(OH)D serum levels and neurodegeneration, as measured by change in serum levels of NfL, in RRMS patients prior to or during IFN-β treatment.

## Data Availability Statement

The datasets generated for this study may be available on request to the corresponding author.

## Ethics Statement

The Regional Committee for Medical and Health Research Ethics in Western Norway and the Norwegian Medicines Agency approved the study protocol, written informed consent was obtain from all patients before study-enrollment and the study was registered at clinicaltrails.gov (NCT00360906).

## Author Contributions

K-MM, ØT, TH, and ER contributed to the conception and the design of the work. JL and ER performed the statistical analysis. ER wrote the first draft of the manuscript. K-MM, ØT, TH, and KV contributed to the acquisition of data for the work. All authors contributed to manuscript revision, and read and approved the submitted version.

## Conflict of Interest

The authors declare that the research was conducted in the absence of any commercial or financial relationships that could be construed as a potential conflict of interest.

## References

[1] Teunissen CE Khalil M. Neurofilaments as biomarkers in multiple sclerosis. Mult Scler. (2012) 18:552–6. 10.1177/1352458512443092 22492131

[2] Novakova L Zetterberg H Sundstrom P Axelsson M Khademi M Gunnarsson M. Monitoring disease activity in multiple sclerosis using serum neurofilament light protein. Neurology. (2017) 89:2230–7. 10.1212/WNL.0000000000004683 29079686 PMC5705244

[3] Kuhle J Nourbakhsh B Grant D Morant S Barro C Yaldizli O. Serum neurofilament is associated with progression of brain atrophy and disability in early MS. Neurology. (2017) 88:826–31. 10.1212/WNL.0000000000003653 28148632 PMC5331872

[4] Disanto G Barro C Benkert P Naegelin Y Schadelin S Giardiello A. Serum Neurofilament light: a biomarker of neuronal damage in multiple sclerosis. AnnNeurol. (2017) 81:857–70. 10.1002/ana.24954 28512753 PMC5519945

[5] Varhaug KN Barro C Bjørnevik K Myhr KM Torkildsen O Wergeland S. Neurofilament light chain predicts disease activity in relapsing-remitting MS. NeurolNeuroimmunolNeuroinflamm. (2018) 5:e422. 10.1212/NXI.0000000000000422 29209636 PMC5707445

[6] Løken-Amsrud KI Holmøy T Bakke SJ Beiske AG Bjerve KS Bjørnarå BT. Vitamin D and disease activity in multiple sclerosis before and during interferon-beta treatment. Neurology. (2012) 79:267–73. 10.1212/WNL.0b013e31825fdf01 22700809

[7] Simpson S Jr Taylor B Blizzard L Ponsonby AL Pittas F Tremlett H. Higher 25-hydroxyvitamin D is associated with lower relapse risk in multiple sclerosis. Ann Neurol. (2010) 68:193–203. 10.1002/ana.22043 20695012

[8] Sandberg L Bistrom M Salzer J Vagberg M Svenningsson A Sundstrom P. Vitamin D and axonal injury in multiple sclerosis. Mult Scler. (2016) 22:1027–31. 10.1177/1352458515606986 26462862

[9] Smolders J Torkildsen O Camu W Holmøy T. An Update on Vitamin D and Disease Activity in Multiple Sclerosis. CNS Drugs. (2019) 33:1187–99. 10.1007/s40263-019-00674-8 31686407 PMC6890630

[10] Holmøy T Røsj,ø E Zetterberg H Blennow K Lindstrøm JC Steffensen LH. Vitamin D supplementation and neurofilament light chain in multiple sclerosis. Acta Neurol Scand. (2019) 139:172–6. 10.1111/ane.13037 30317548

[11] Smolders J Mimpen M Oechtering J Damoiseaux J van den Ouweland J Hupperts R. Vitamin D3 supplementation and neurofilament light chain in multiple sclerosis. Acta Neurol Scand. (2020) 141:77–80. 10.1111/ane.13185 31657006

[12] Holick MF Vitamin D: the underappreciated D-lightful hormone that is important for skeletal and cellular health. Curr Opin Endocrinol Diabetes Obesity. (2002) 9:87–98 10.1097/00060793-200202000-00011

[13] Torkildsen O Wergeland S Bakke S Beiske AG Bjerve KS Hovdal H. omega-3 fatty acid treatment in multiple sclerosis (OFAMS Study): a randomized, double-blind, placebo-controlled trial. ArchNeurol. (2012) 69:1044–51. 10.1001/archneurol.2012.283 22507886

[14] Kuhle J Barro C Andreasson U Derfuss T Lindberg R Sandelius A. Comparison of three analytical platforms for quantification of the neurofilament light chain in blood samples: ELISA, electrochemiluminescence immunoassay and Simoa. Clin Chem Lab Med. (2016) 54:1655–61 10.1515/cclm-2015-1195 27071153

[15] Ascherio A Munger KL White R Kochert K Simon KC Polman CH. Vitamin D as an early predictor of multiple sclerosis activity and progression. JAMA Neurol. (2014) 71:306–14. 10.1001/jamaneurol.2013.5993 24445558 PMC4000029

[16] Smolders J Moen SM Damoiseaux J Huitinga I Holmøy T. Vitamin D in the healthy and inflamed central nervous system: access and function. J Neurol Sci. (2011) 311:37–43. 10.1016/j.jns.2011.07.033 21862439

[17] Røsjø E Myhr KM Løken-Amsrud KI Bakke SJ Beiske AG Bjerve KS. Vitamin D status and effect of interferon-beta1a treatment on MRI activity and serum inflammation markers in relapsing-remitting multiple sclerosis. J Neuroimmunol. (2015) 280:21–8. 10.1016/j.jneuroim.2015.02.001 25773151

[18] Enzinger C Fuchs S Pichler A Wallner-Blazek M Khalil M Langkammer C. Predicting the severity of relapsing-remitting MS: the contribution of cross-sectional and short-term follow-up MRI data. MultScler. (2011) 17:695–701. 10.1177/1352458510394454 21228028

[19] Zivadinov R Treu CN Weinstock-Guttman B Turner C Bergsland N O'Connor K. Interdependence and contributions of sun exposure and vitamin D to MRI measures in multiple sclerosis. J Neurol Neurosurg Psychiatry. (2013) 84:1075–81. 10.1136/jnnp-2012-304661 23385850

