# Supplementary material for: Natural Variation of Vitamin D and Neurofilament Light Chain in Relapsing-Remitting Multiple Sclerosis
Source: Front Neurol. 2020 Apr 30;11:329. doi: 10.3389/fneur.2020.00329 (PMC7205013; doi:10.3389/fneur.2020.00329)
Supplement: Supplementary file 1 [file Data_Sheet_1.docx]

**Supplemental table 1. Serum 25(OH)D and NfL levels during the study**

| Study month | NfL (pg/mL) | | | 25(OH)D (nmol/L) | | |
| --- | --- | --- | --- | --- | --- | --- |
|  | Samples (N) | Mean (SD) | *p*-value  0 vs 24* | Samples (N) | Mean (SD) | *p*-value  0 vs 24* |
| 0 | 85 | 40.2 (22.4) | <0.01 | 87 | 61.2 (19.3) | 0.29 |
| 3 | 84 | 38.5 (22.9) |  | 87 | 69.1 (26.0) |  |
| 6 | 85 | 36.5 (18.6) |  | 88 | 75.1 (27.6) |  |
| 12 | 82 | 32.8 (18.7) |  | 87 | 66.6 (24.7) |  |
| 24 | 79 | 28.2 (12.5) |  | 85 | 63.6 (21.8) |  |

N: Number; SD: Standard deviation *Calculated with a paired sample *t*-test

**Supplemental table 2. Serum 25(OH)D and NfL levels during the whole study stratified by calendar month**

| Calendar month | NfL (pg/mL) | | 25(OH)D (nmol/L) | |
| --- | --- | --- | --- | --- |
|  | Samples (N) | Mean (SD) | Samples (N) | Mean (SD) |
| 1 | 24 | 38.0 (20.0) | 55 | 60.2 (17.2) |
| 2 | 40 | **43.5 (27.7)** | 69 | 59.5 (19.5) |
| 3 | 51 | 32.1 (13.1) | 84 | **50.3 (16.8)** |
| 4 | 22 | 34.6 (20.6) | 41 | 53.5 (20.9) |
| 5 | 59 | 38.1 (24.5) | 75 | 63.3 (19.5) |
| 6 | 47 | 31.7 (15.1) | 88 | 72.9 (21.9) |
| 7 | 20 | 35.3 (27.1) | 41 | 84.8 (25.6) |
| 8 | 40 | 39.6 (21.8) | 73 | **102.3 (33.7)** |
| 9 | 26 | **28.9 (11.6)** | 61 | 84.7 (23.1) |
| 10 | 16 | 33.7 (13.4) | 46 | 72.1 (26.4) |
| 11 | 23 | 37.4 (16.5) | 59 | 71.1 (26.8) |
| 12 | 47 | 30.7 (14.0) | 88 | 61.4 (20.4) |

N: Number; SD: Standard deviation

**Supplemental table 3. Serum 25(OH)D and NfL levels prior to IFN-β therapy stratified by calendar month**

| Calendar month | NfL (pg/mL) | | 25(OH)D (nmol/L) | |
| --- | --- | --- | --- | --- |
|  | Samples (N) | Mean (SD) | Samples (N) | Mean (SD) |
| 1 | 12 | 40.8 (21.0) | 25 | 59.1 (14.6) |
| 2 | 14 | **54.8 (31.6)** | 21 | 55.8 (19.1) |
| 3 | 28 | 35.9 (12.3) | 43 | 55.4 (18.3) |
| 4 | 11 | 38.0 (24.6) | 21 | **52.4 (24.2)** |
| 5 | 33 | 44.1 (29.3) | 41 | 61.5 (19.1) |
| 6 | 29 | 34.8 (16.9) | 48 | 68.1 (20.3) |
| 7 | 18 | 37.5 (27.7) | 23 | 86.0 (25.0) |
| 8 | 33 | 42.0 (22.9) | 36 | **93.2 (28.8)** |
| 9 | 20 | **30.5 (11.0)** | 24 | 77.6 (23.4) |
| 10 | 10 | 31.1 (11.0) | 14 | 71.4 (29.9) |
| 11 | 20 | 37.2 (14.3) | 21 | 65.3 (20.7) |
| 12 | 26 | 33.9 (15.9) | 31 | 59.7 (18.4) |

N: Number; SD: Standard deviation

**Supplemental table 4. Serum 25(OH)D and NfL levels during IFN-β therapy stratified by calendar month**

| Calendar month | Samples (N) | NfL (pg/mL) | 25(OH)D (nmol/L) | |
| --- | --- | --- | --- | --- |
|  |  | Mean (SD) | Samples (N) | Mean (SD) |
| 1 | 12 | 35.2 (19.4) | 30 | 61.2 (19.2) |
| 2 | 26 | 37.5 (23.9) | 48 | 61.1 (19.7) |
| 3 | 23 | 27.5 (12.7) | 41 | **44.9 (13.2)** |
| 4 | 11 | 31.2 (16.0) | 20 | 54.8 (17.3) |
| 5 | 26 | 30.3 (13.6) | 34 | 65.4 (20.0) |
| 6 | 18 | 26.7 (10.1) | 40 | 78.7 (22.6) |
| 7 | 2 | **15.4 (7.7)** | 18 | 83.2 (26.9) |
| 8 | 7 | 28.2 (10.3) | 37 | **111.1 (36.1)** |
| 9 | 6 | 23.8 (13.1) | 37 | 89.2 (22.1) |
| 10 | 6 | 38.1 (16.8) | 32 | 72.4 (25.2) |
| 11 | 3 | **38.6 (32.4)** | 38 | 74.2 (29.4) |
| 12 | 21 | 26.7 (10.0) | 57 | 62.4 (21.5) |

N: Number; SD: Standard deviation

**Supplemental table 5. Change in serum NfL concentration with every 10 nmol/L increase of 25(OH)D**

| Study period | % (CI)* | *p*-value* |
| --- | --- | --- |
| Before IFN-β | 1.2 (-0.4 - 2.7) | 0.14 |

IFN-β: Interferon beta-1a; CI: 95% confidence interval *Calculated with linear regression models with random intercepts for each patient and inclusion of 25(OH)D measurements paired with NfL measurements three months later

**Supplemental figure. Baseline serum NfL and 25(OH)D levels**


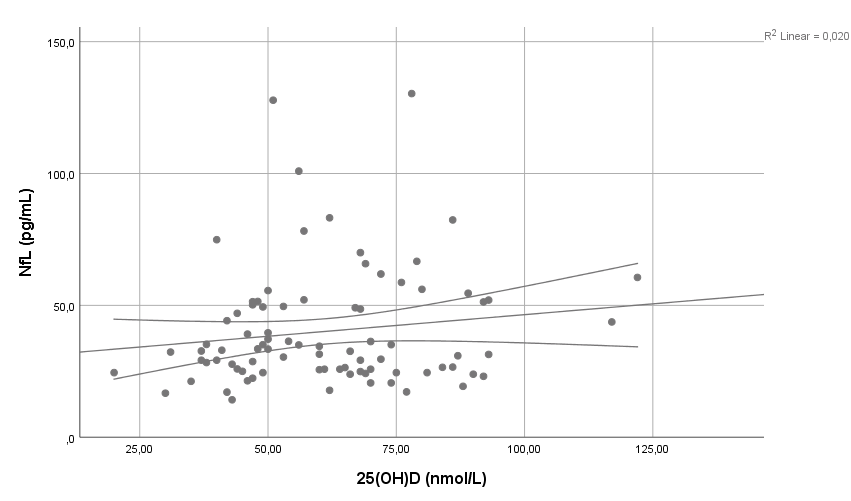


Correlation between baseline serum NfL and 25(OH)D concentrations (Pearson's correlation coefficient (r) of 0.124, *p*-value=0.19)
